# Fast Ca^2+^ Transients of Inner Hair Cells Arise Coupled and Uncoupled to Ca^2+^ Waves of Inner Supporting Cells in the Developing Mouse Cochlea

**DOI:** 10.3389/fnmol.2018.00264

**Published:** 2018-07-30

**Authors:** Tobias Eckrich, Kerstin Blum, Ivan Milenkovic, Jutta Engel

**Affiliations:** ^1^Department of Biophysics, Center for Integrative Physiology and Molecular Medicine (CIPMM), Saarland University, School of Medicine, Homburg, Germany; ^2^Carl-Ludwig-Institute for Physiology, Faculty of Medicine, University of Leipzig, Leipzig, Germany

**Keywords:** cochlea, spontaneous activity, development, Ca^2+^ wave, inner hair cell, Ca_V_1.3

## Abstract

Before the onset of hearing, which occurs around postnatal day 12 (P12) in mice, inner hair cells (IHCs) of the immature cochlea generate sound-independent Ca^2+^ action potentials (APs), which stimulate the auditory pathway and guide maturation of neuronal circuits. During these early postnatal days, intercellular propagating Ca^2+^ waves elicited by ATP-induced ATP release are found in inner supporting cells (ISCs). It is debated whether IHCs are able to fire Ca^2+^ APs independently or require a trigger by an ISC Ca^2+^ wave. To identify the Ca^2+^ transients of IHCs underlying Ca^2+^ APs and to analyze their dependence on ISC Ca^2+^ waves, we performed fast Ca^2+^ imaging of Fluo-8 AM-loaded organs of Corti at P4/P5. Fast Ca^2+^ transients (fCaTs) generated by IHCs were simultaneously imaged with Ca^2+^ waves in ISCs. ISC Ca^2+^ waves frequently evoked bursts consisting of >5 fCaTs in multiple adjacent IHCs. Although Ca^2+^ elevations of small amplitude appeared to be triggered by ISC Ca^2+^ waves in IHCs of Ca_v_1.3 knockout mice we never observed fCaTs, indicating their requirement for Ca^2+^ influx through Ca_v_1.3 channels. The Ca^2+^ wave-triggered Ca^2+^ upstroke in wildtype IHCs occurred 0.52 ± 0.27 s later than the rise of the Ca^2+^ signal in the adjacent ISCs. In comparison, superfusion of 1 μM ATP elicited bursts of fCaTs in IHCs starting 0.99 ± 0.34 s prior to Ca^2+^ elevations in adjacent ISCs. PPADS irreversibly abolished Ca^2+^ waves in ISCs and reversibly reduced fCaTs in IHCs indicating differential involvement of P2 receptors. IHC and ISC Ca^2+^ signals were however unaltered in P2X2R/P2X3R double knockout or in P2X7R knockout mice. Together, our data revealed a fairly similar occurrence of fCaTs within a burst (56.5%) compared with 43.5% as isolated single fCaTs or in groups of 2–5 fCaTs (minibursts). We provide evidence that IHCs autonomously generate single fCaTs and minibursts whereas bursts synchronized between neighboring IHCs were mostly triggered by ISC Ca^2+^ waves. Neonatal IHCs thus spontaneously generate electrical and Ca^2+^ activity, which is enhanced and largely synchronized by activity of ISCs of Kölliker’s organ indicating two sources of spontaneous activity in the developing auditory system.

## Introduction

Inner Hair Cells (IHCs) generate Ca^2+^ action potentials (APs) in the absence of sound before the onset of hearing, which in mice occurs around postnatal day 12 (P12). These APs translate into release of glutamate (Kros et al., 1998; Brandt et al., 2003, 2007; Marcotti et al., 2003b; Johnson et al., 2007, 2011, 2013; Sendin et al., 2014), initiate excitatory postsynaptic potentials and APs in spiral ganglion neurons (Glowatzki and Fuchs, 2002) and activate the afferent auditory pathway (Tritsch et al., 2010; Clause et al., 2014). Experience-independent structured electrical activity is a common phenomenon in developing sensory systems, thought to contribute to maturation of synapses, ion channel expression and refinement of neuronal networks (Shatz, 1996; Stellwagen and Shatz, 2002; Moody and Bosma, 2005; Blankenship and Feller, 2010; Leighton and Lohmann, 2016).

It is debated whether IHCs generate Ca^2+^ APs autonomously (Kros et al., 1998; Brandt et al., 2003, 2007; Marcotti et al., 2003b; Johnson et al., 2007, 2011, 2013, 2017; Sendin et al., 2014) or require Ca^2+^ waves of inner supporting cells (ISCs) as a trigger (Tritsch et al., 2007, 2010; Tritsch and Bergles, 2010; Wang et al., 2015). The immature cochlea contains Kölliker’s organ, which harbors tall cylindrical cells that spontaneously generate intercellular Ca^2+^ waves (Tritsch et al., 2007; Anselmi et al., 2008; Majumder et al., 2010; Mammano and Bortolozzi, 2018). Wave propagation is sustained by ATP-dependent ATP release: extracellular ATP releases intracellular Ca^2+^ through a P2Y receptor-IP_3_-dependent mechanism. In turn, the rise in [Ca^2+^]_i_ increases the open probability of connexin hemichannels for ATP, which are located at the endolymphatic surface of the ISCs (Tritsch et al., 2007; Anselmi et al., 2008; Majumder et al., 2010; Mammano and Bortolozzi, 2018). IP_3_ may also contribute to wave propagation by diffusing through gap junctions to ISC neighbors (Anselmi et al., 2008; Majumder et al., 2010; Ceriani et al., 2016b).

ISC Ca^2+^ waves may trigger Ca^2+^ APs in IHCs by two potential mechanisms: (i) ATP-induced depolarization to the threshold for regenerative APs (Tritsch et al., 2007; Johnson et al., 2011) mediated by P2X2R, P2X3R and P2X7R (Brändle et al., 1999; Nikolic et al., 2003; Housley et al., 2006; Huang et al., 2010) or (ii) [Ca^2+^]_i_-dependent activation of TMEM-16A channels in ISCs that allow Cl^−^ efflux followed by efflux of K^+^ and water (Wang et al., 2015). The resulting increase in extracellular K^+^ may sufficiently depolarize IHCs to evoke Ca^2+^ APs. Accordingly, a Ca^2+^ wave in the ISC compartment would elicit synchronized bursts of Ca^2+^ APs in neighboring IHCs (Wang et al., 2015).

Although IHC Ca^2+^ APs, which are based on regenerative opening of Ca_v_1.3 Ca^2+^ channels, K_v_ and SK2 K^+^ channels, have been analyzed in depth by patch-clamp recordings (Brandt et al., 2003; Marcotti et al., 2003b; Johnson et al., 2007, 2011, 2013, 2017; Sendin et al., 2014) the accompanying Ca^2+^ transients in IHCs have not been studied so far. Moreover, we still lack simultaneous imaging data resolving the temporal and spatial correlation between fast Ca^2+^ transients in IHCs and Ca^2+^ waves in ISCs. We therefore conducted Ca^2+^ imaging in the immature mouse organ of Corti to determine whether Ca^2+^ transients in IHCs occur independent of ISC Ca^2+^ waves.

## Materials and Methods

### Animals

All experimental procedures were conducted in agreement with the European Communities Council Directive (2010/63/EU) in accordance with the German law on the use of laboratory animals and were approved by the Saxonian District Government, Leipzig (TVV59/16 and T34/16), and the regional board for scientific animal experiments of the Saarland. Prehearing mice (P4–P6) of either sex were studied. NMRI mice (Charles River, Sulzfeld, Germany and bred in-house; 64 animals) were used unless stated otherwise and are designated as controls. Ca_v_1.3^−/−^ mice (Platzer et al., 2000), which were bred to either C57BL6/N (seven animals) or NMRI background (13 animals) for >10 generations, mice deficient for the purinergic receptor P2X7 (P2X7R^−/−^ mice; The Jackson Laboratories, Bar Harbor, ME, USA; 21 animals) and P2X2R/P2X3R double knockout mice (P2X2R^−/−^/P2X3R^−/−^; 20 animals; Cockayne et al., 2005) both on C57BL6/N background were used. Every experimental observation was confirmed in at least three experiments using different animals.

### Tissue Preparation

**Figure 1:**
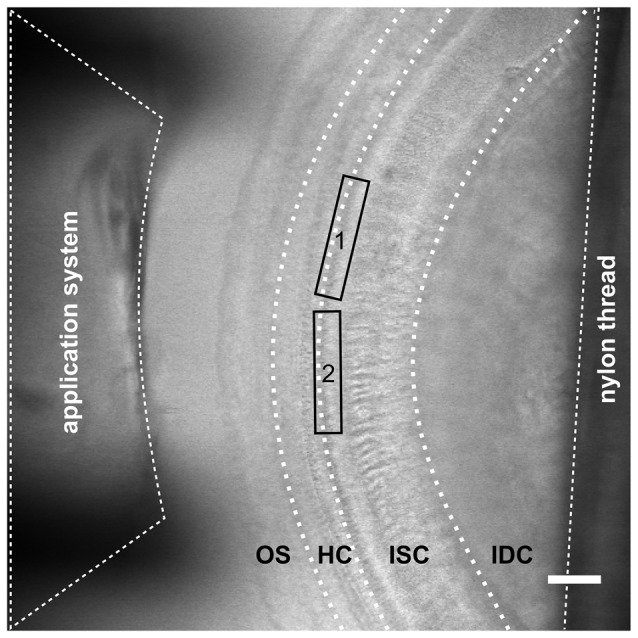
Configuration for imaging inner supporting cell (ISC) Ca^2+^ waves and inner hair cell (IHC) Ca^2+^ transients in the immature cochlea of the mouse. Transmitted light image of an acutely dissected whole-mount preparation of the apical turn from a P4 control mouse organ of Corti. The focus is set on the hair cell region (HC) with three rows of outer hair cells and one row of IHCs and the great epithelial ridge with the ISCs. Because of the upward slope of the epithelium towards the modiolus the outer sulcus region (OS) and interdental cells (IDCs) are out of focus. The outlet of a gravity-fed application system made from tapered PVC tubing with an inner diameter of 400 μm was placed at the outer sulcus (pillar) side for gentle application of solutions with blockers or agonists. The tissue was held by two soft nylon threads (one is visible as the rightmost black shadow) fixed to a stainless steel ring that fit into a recording chamber. Care was taken that only the outermost edges of the tissue were held down to the glass bottom of the chamber. One to two recordings were made per apical cochlear turn. Recording frames are depicted by the rectangles labeled 1 and 2. The distance from the thread to the recording site was >200 μm. Scale bar, 50 μm.

**Figure 2:**
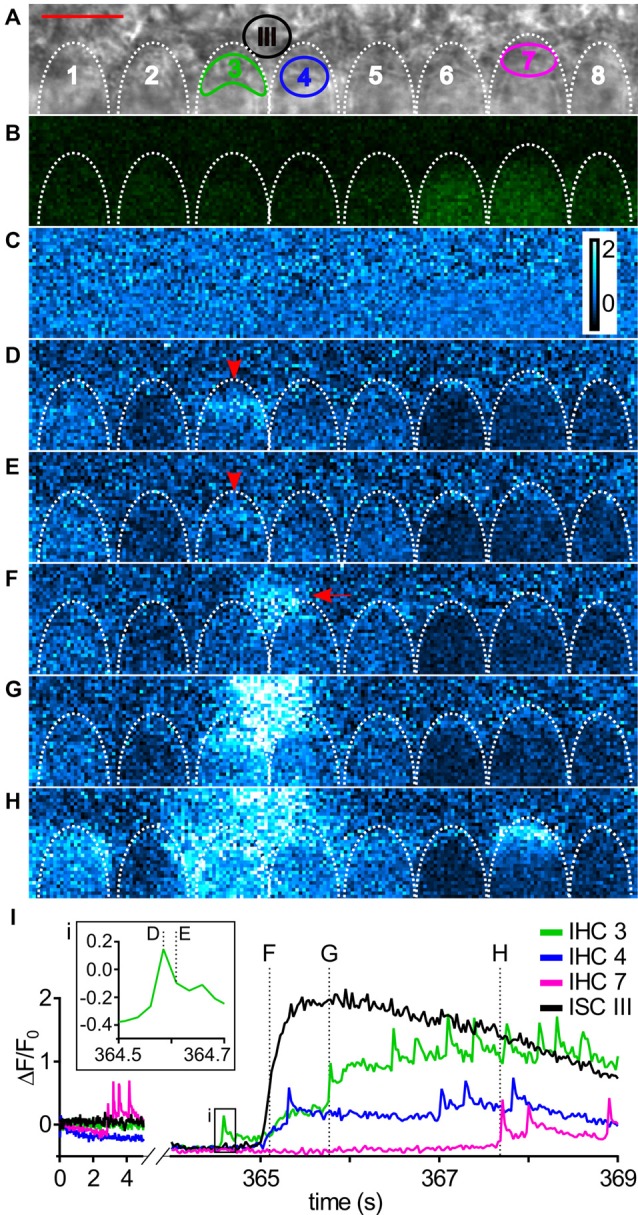
Imaging Ca^2+^ waves and transients in ISCs and IHCs in the immature cochlea of the mouse.** (A)** Transmitted light image of the scan field in the organ of Corti of a control mouse (P5) including basolateral IHC poles and adjacent ISCs at high resolution (pixel size 41.6 nm × 41.6 nm). Scale bar, 10 μm. Shapes of basolateral poles of IHCs are outlined by white dotted lines; regions of interest (ROIs) in IHCs 3, 4 and 7 and in ISC III close to IHCs 3 and 4 are indicated by colors. **(B)** Raw fluorescence of the Fluo-8 AM-loaded specimen (green) at *t* = 0 s with IHCs 6–8 showing slightly elevated [Ca^2+^]_i_ at *t* = 0 s. To allow for faster scanning, pixel size was increased to 416 nm × 416 nm compared with **(A)**. **(C–H)** Color-coded relative fluorescence changes Δ*F*/*F*_0_ = (*F*(*t*) − *F*_0_)/*F*_0_ with *F*_0_ being the average of the first 10 images starting at *t* = 0 s; color scale bar indicates a range of 0–2 **(C)**. Δ*F*/*F*_0_ images are shown at 0 s and in (D–H) at the time points indicated by dashed lines in (I). **(D,E)** The basal pole of IHC 3 showed an isolated fast transient (arrowhead) that was not triggered by an ISC Ca^2+^ wave. **(F–H)** ISC III close to IHC 3 and IHC 4 displayed a localized Ca^2+^ transient (F, arrow) that spread as a Ca^2+^ wave towards the IHCs (G,H). **(H)** 2.6 s after start of the Ca^2+^ wave (see F), IHCs 1, 3, 4, 5, 7 showed elevated Ca^2+^, whereas IHCs 2, 6 and 8 did not. **(I)** Intracellular Ca^2+^ changes as Δ*F*/*F*_0_ in ROIs of IHCs 3, 4 and 7 (colored) and ISC III (black). Time = 0 s corresponds to image **(C)**; time points of images (D–H) are indicated by dotted lines, with time points of (D,E) shown in the inset (i). IHCs displayed Ca^2+^ transients with a faster upstroke than ISCs shown for ISC III. Scale bar: 10 μm. Data file 20150114_036, frame rate = 41 Hz (scan time of single frame = 24.4 ms), 20×/1.0 NA objective.

Neonatal mice were sacrificed by decapitation. Cochleae were immediately dissected in ice-cold physiological solution mimicking perilymph (in mM): 143 NaCl, 5.8 KCl, 1.3 CaCl_2_, 0.9 MgCl_2_, 5.6 glucose, 10 HEPES and 0.9 NaH_2_PO_4_, 305 mOsm/l, pH 7.35. The organ of Corti was dissected out of the cochlea with great care to preserve the integrity of the tissue. The apical turn was cut and placed in a recording chamber underneath a soft thread of a nylon mesh, which was attached to a steel ring (Figure 1). Nylon threads had an average distance to the recording window of 253 ± 47 μm (determined for 26 recordings). After cautiously removing the tectorial membrane, the specimen was incubated in the perilymph-like solution containing 10 μM of the cell-permeable Ca^2+^ indicator Fluo-8^®^ AM (AAT Bioquest Inc., Sunnyvale, CA, USA) that had been dissolved in a Pluronic-DMSO mixture (100 mg/ml; Thermo Fisher Scientific Inc., Waltham, MA, USA). To allow for uptake of Fluo-8 AM and hydrolysis, the recording chamber with the tissue was kept in a dark, humid plastic box for 40 min at room temperature. The chamber was mounted on the stage of the upright laser scanning microscope LSM 710 (Zeiss Microscopy GmbH, Göttingen, Germany) and perfused with perilymph-like solution for at least 7 min to remove any Fluo-8 AM prior to the experiment. Only healthy looking epithelia with smooth surfaces, absence of vacuoles and low background fluorescence in both IHCs and ISCs were used for imaging (Figure 2A). IHCs were recorded within a fractional distance of 10–25% from the apex, which corresponds to a frequency range of about 7–10 kHz in the adult animal (Müller et al., 2005). Agonists and antagonists (ATP; 1 or 10 μM; PPADS; 100 μM; both Sigma-Aldrich, St. Louis, MO, USA) were diluted in perilymph-like solution (see above) and applied by a gravity-driven custom-made application system with a multi-barreled pipette (Figure 1), which led to a change of the solution above ISCs and IHCs within 3–6 s. Unless stated otherwise, the specimen was constantly superfused with either perilymph-like solution, the same solution containing the respective agonist/antagonist, or with Ca^2+^-free solution, respectively, via the application system at 0.29 ml/min. To avoid movement artifacts caused by changes in fluid pressure, the switch between two solutions was performed by simultaneously closing respectively opening the valves of the respective reservoirs. The Ca^2+^-free solution containing the following (in mM): 143 NaCl, 5.8 KCl, 2.2 MgCl_2_, 5.6 glucose, 10 HEPES, 0.9 NaH_2_PO_4_ and 0.5 EGTA acid, 305 mOsm/l, pH 7.35. The distance between the scan field and the lateral edges of the tissue combined with the application of perilymph-like solution without/with agonist/antagonists from the pillar side prevented spillover of ATP from potentially damaged cells and ISCs. The delay in solution flow from reaching the IHCs to reaching the ISCs was estimated to be <20 ms.

### Ca^2+^ Imaging, Analysis and Data Handling

Data were recorded using a LSM 710 based on an upright AxioExaminer microscope with 40×/1.0 NA or 20×/1.0 NA Plan Apochromat water immersion objectives and Zen-2012 SP1 (Black edition) software (all Zeiss). Fluo-8 fluorescence was excited with the 488 nm line of an argon laser. Laser intensity was set to very low levels to allow for long continuous recordings (>10 min, up to 30 min) while avoiding photobleaching and strong phototoxicity. We opened up the pinhole to maximal seven airy units accepting a reduced spatial resolution in favor of increased fluorescence intensity at the chosen low laser power and a higher scan speed. Recordings were performed at room temperature (21°C ± 1°C). Because Fluo-8 based on Fluo-4 (Gee et al., 2000) is a non-ratiometric Ca^2+^ indicator with a high quantum yield and a *K*_d_ of 389 nM we determined the relative fluorescence changes $\text{Δ}\frac{F}{F_{0}}$ corresponding to [Ca^2+^]_i_ changes in ISCs and IHCs off-line:

(1) $$\text{Δ}\frac{F(t)}{F_{0}}=\frac{F(t)-F_{0}}{F_{0}}=\frac{\text{Δ}F}{F_{0}}$$

where *F*(*t*) is the fluorescence intensity of a pixel at a given time *t* and *F*_0_ the fluorescence of this pixel averaged over the first 10 images without obvious Ca^2+^ changes.

For imaging the spread of ISC Ca^2+^ waves in Kölliker’s organ, scan fields of about 200 μm × 100 μm were chosen resulting in frame rates of ≤5 Hz (scan times >200 ms). To resolve the much faster fCaTs in IHCs, we increased the frame rate by: (i) imaging smaller scan fields; and (ii) increasing the pixel size to 0.4 μm × 0.4 μm (up to 0.6 μm × 0.6 μm). Scan fields were 140 μm ± 21 μm in width (axis parallel to the IHCs) and 27 μm ± 5 μm in height (Figures 1, 2A) and comprised the basolateral poles of 7–9 (up to 23) IHCs with a stripe of ISCs extending up to 36 μm in the modiolar direction. This led to frame rates of >31 Hz and to corresponding shorter scan times of ≤32 ms (except Figure 6, 36.2 ms), which allowed simultaneous imaging of Ca^2+^ waves in ISCs and fCaTs in IHCs. For achieving an even better temporal resolution of fCaTs, a subset of control IHCs was imaged at 175 Hz (scan time of a single frame: 5.73 ms). With these settings, photobleaching amounted to 0.5 Δ*F*/*F*_0_ at maximum during periods of ≥6 min. Offline analysis and graphical representation was performed using FIJI (Schindelin et al., 2012), IGOR 6.12 (Wavemetrics, Inc., Lake Oswego, OR, USA) and Inkscape™ 0.91. Regions of interest (ROIs) in a time series (*t*-series) of fluorescence images were defined by activity patterns within IHCs and ISCs and were validated using a high-resolution transmitted light image acquired prior to the *t-series* with a 100-fold smaller pixel area. Transmitted light images were also taken after a *t-series* to check for a potential tissue drift. Data are reported as mean ± standard deviation (SD) except data in Figure 10B, where mean ± SEM is given.

**Figure 3:**
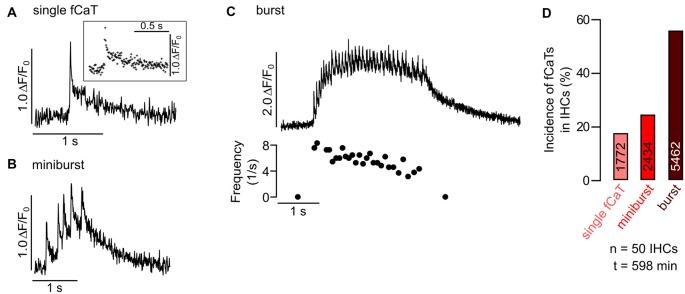
Classification of IHC fast Ca^2+^ transients (fCaTs) at high temporal resolution. **(A–C)** Types of fCaTs as Δ*F*/*F*_0_ of an IHC at 175 Hz acquisition rate. **(A)** A single fCaT of an IHC with the inset depicting individual data points. **(B)** A miniburst with five fCaTs of the same IHC. **(C)** A burst (>5 fCaTs, top) and the corresponding interspike intervals (bottom). Data file: 20140614_031, frame rate = 175 Hz (scan time of single frame = 5.73 ms), 40×/1.0 NA objective. **(D)** Relative incidence of different types of fCaTs—single events, fCaTs occurring in minibursts or in bursts recorded in 50 IHCs from four mice at standard scan rate (~30 Hz). Total numbers of fCaTs analyzed for the different types of IHC activity are given in the bars.

**Figure 4:**
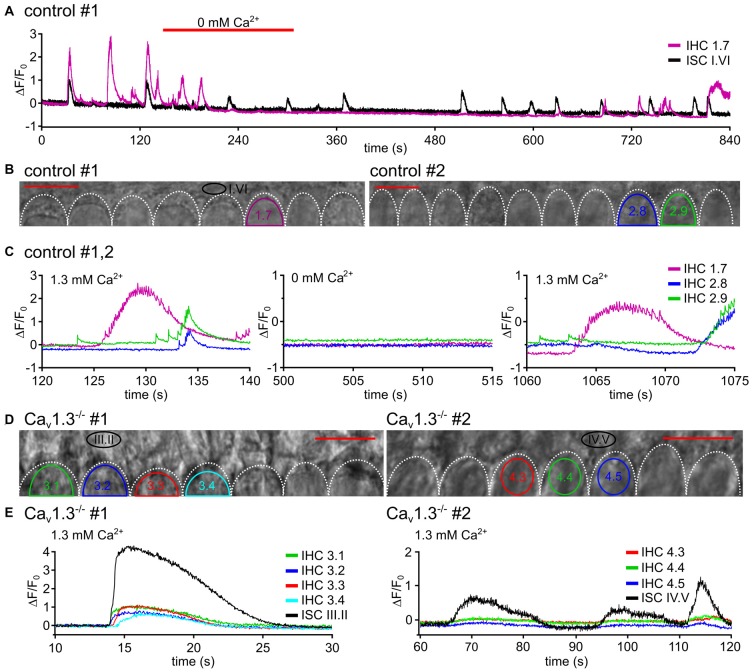
Fast Ca^2+^ transients require influx of extracellular Ca^2+^ through Ca_v_1.3 channels. **(A)** In a P5 control specimen (#1), the effects of superfusion with nominally Ca^2+^-free solution on [Ca^2+^]_i_ changes are shown for the ROIs of one IHC (IHC 1.7) and one ROI of adjacent ISCs (A; for transmitted light image see B, left). Red bars above the trace indicate superfusion with Ca^2+^-free solution. Here, the application pipette was oriented from the lateral side of the organ of Corti (different from the position in Figure 1) to allow for flow of the 0 mM Ca^2+^ solution underneath the reticular lamina to the IHCs’ basolateral poles. The solution needed 3–6 s for overcoming the dead space in the tubing plus about 50 s to reach the IHCs at the recording site. IHC [Ca^2+^]_i_ elevations were inhibited or strongly suppressed, whereas Ca^2+^ transients of the ROI ISC I.VI were still present (black trace). **(B)** Transmitted light images of the scan fields of two organs of Corti (control #1 and control #2) of 5 day-old control mice including the basolateral poles of IHC and adjacent ISCs at high resolution (pixel size 41.6 nm × 41.6 nm), respectively. Frame rate 31.2 Hz (single frame = 32 ms). **(C)** Δ*F*/*F*_0_ of IHC 1.7 (from A,B, left) from specimen control #1 and of IHCs 2.8 and 2.9 from control #2 (B, right), which was treated in the same way, at larger time resolution showed single or burst-like fCaTs, partially on top of elevated [Ca^2+^]_i_, in perilymph-like (1.3 mM Ca^2+^) solution (left). Nominally Ca^2+^-free solution abolished fCaTs (middle). After switching back to 1.3 mM Ca^2+^, single or burst-like fCaTs re-appeared.** (D)** Transmitted light images of the scan fields of two organs of Corti of 4-day-old Ca_v_1.3^−/−^ mice (#1, #2) including the basolateral poles of IHCs and adjacent ISCs at high resolution (pixel size 41.6 nm × 41.6 nm), respectively. **(E)** Despite Ca^2+^ waves reaching the IHC region, fCaTs were absent from IHCs of 4-day-old Ca_v_1.3^−/−^ mice at normal extracellular Ca^2+^ (1.3 mM). Whereas ISC Ca^2+^ waves frequently elicited long-lasting small increases in Δ*F*/*F*_0_ in IHCs that resembled the down-scaled ISC Ca^2+^ increase and lacked any fCaT (left, animal #1, ISC III.II, black trace; IHC 1–IHC 4, colored traces), on rare occasions waves did not elicit any response in IHCs of Ca_v_1.3^−/−^ mice (right, animal #2). Scale bars, 10 μm, frame rates 32.2 Hz (single frame = 31.1 ms).

**Figure 5:**
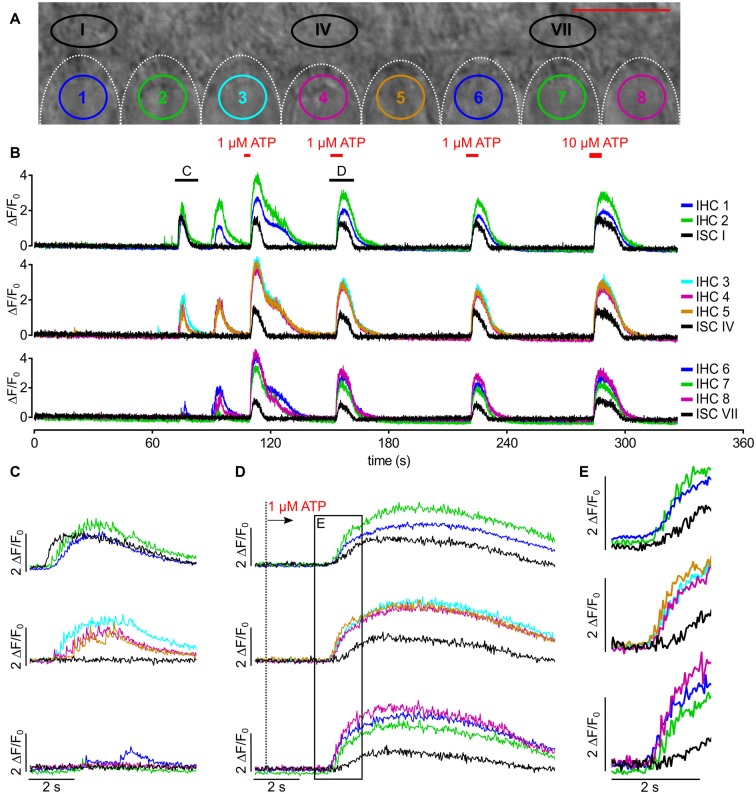
Synchronized [Ca^2+^]_i_ bursts of adjacent IHCs are mediated by invading ISC Ca^2+^ waves or by superfusion of ATP. **(A)** Transmitted light image of the scan field in the organ of Corti of a control mouse (P5) including basolateral poles of IHC and adjacent ISCs at high resolution (pixel size 41.6 nm × 41.6 nm). Scale bar, 10 μm. **(B)** Overview of spontaneous and ATP-evoked changes in Δ*F*/*F*_0_ in ROIs of all IHCs and three ROIs of their adjacent modiolar ISCs during 5 min 30 s recording time. Black bars on top of panel (B) indicate intervals that are displayed with enhanced temporal resolution in (C,D); superfusion with 1 or 10 μM ATP is indicated at the top. **(C)** Spontaneous activity without external application of ATP. A Ca^2+^ transient in ISC I induced a Ca^2+^ wave that triggered fCaTs in IHCs, which appeared to be synchronized between IHC 1 to IHC 5. IHCs 6 and 7 responded with a delay of >1 s and only with one and four fCaTs, respectively. **(D)** One micromolar ATP superfused from the pillar side induced Ca^2+^ elevations in all IHCs and all ISCs. The dotted line depicts the switch from control solution to extracellular solution containing 1 μM ATP. **(E)** Enlargement of the box in (D) shows that the increase in Δ*F*/*F*_0_ of IHCs preceded that of the ISCs. Data file 20150807_041, sampling frequency = 31.9 Hz (single frame = 31.3 ms), 20×/1.0 NA objective.

**Figure 6:**
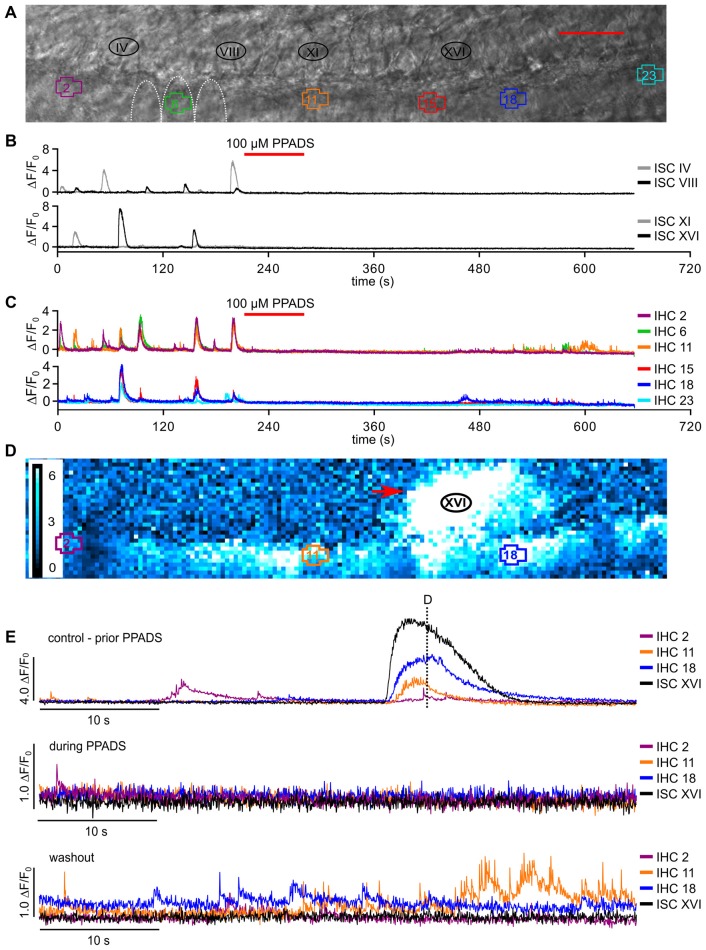
Block of P2 receptors by PPADS irreversibly abolishes Ca^2+^ waves and transients in ISCs and reversibly reduces fast Ca^2+^ transients in IHCs. **(A)** Transmitted light image of the scan field in the organ of Corti of a P5 control mouse including basolateral IHC poles and adjacent ISCs at high resolution (pixel size: 65 nm × 65 nm). ROIs of IHCs are numbered 1 to 23, and four ROIs of adjacent ISCs are indicated (IV, VIII, XI and XVI, which are close to the IHCs with the Arabic number counterpart). For clarity only three IHCs (5–7) are outlined by white dotted lines; scale bar = 20 μm. (B,C) Overview of Δ*F*/*F*_0_ in six selected IHCs **(C)** and selected adjacent ISCs **(B)** during 11 min recording time. The red bar on top of panels (B,C) indicates perfusion of 100 μM PPADS-containing solution (red). **(D)** Color-coded relative fluorescence changes Δ*F*/*F*_0_ at *t* = 72.5 s; a typical Ca^2+^ wave (red arrow) prior to PPADS application is shown at approximately maximum spread. **(E)** Δ*F*/*F*_0_ for selected IHCs and for ISC XVI (adjacent to IHC 16, black trace) at larger time resolution before superfusion with PPADS (top, control condition, dashed line refers to the time point shown in D). Middle panel: 100 μM PPADS blocked slow Ca^2+^ elevations in all ISCs, shown for ISC XVI, and in IHCs. In few IHCs, sparse fCaTs were still present (IHC 2, green trace). Whereas washout of PPADS for ≥5 min restored fCaTs in many IHCs, this was not the case for Ca^2+^ elevations in ISCs, shown for ISC XVI (bottom). Data file 2015021_013, frame rate = 27.6 Hz (single frame = 36.2 ms), 20×/1.0 NA objective.

**Figure 7:**
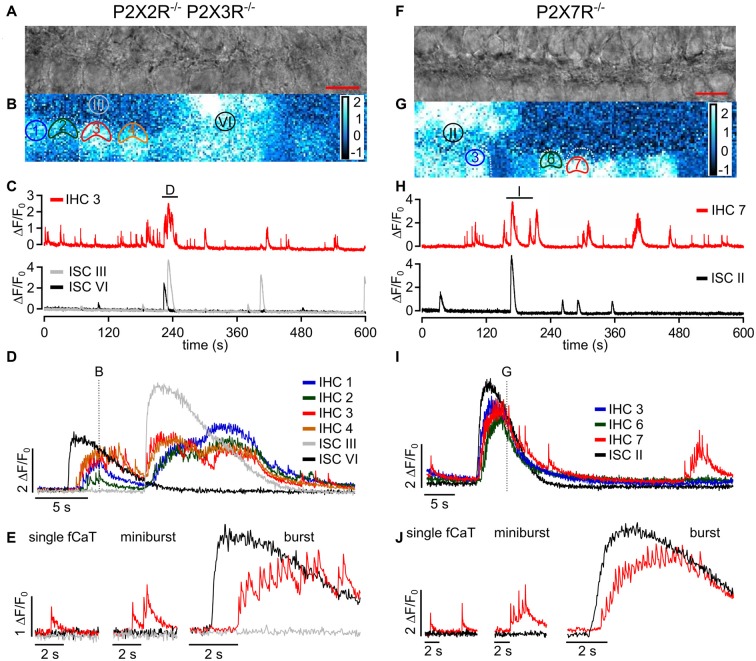
ISC Ca^2+^ waves and IHC fCaTs are present in epithelia of P2X2R^−/−^/P2X3R^−/−^ and P2X7R^−/−^ mice. **(A)** Transmitted light image of the scan field in the organ of Corti of a P2X2R^−/−^/P2X3R^−/−^ mouse aged P4 including basolateral IHC poles and adjacent ISCs at high resolution (pixel size 51 nm × 51 nm; scale bar = 10 μm). **(B)** A typical Ca^2+^ wave, which originated in ISC VI, is shown as color-coded relative fluorescence changes Δ*F*/*F*_0_ at approximately maximum spread. Note that almost all IHCs showed elevated Ca^2+^ levels. ROIs of selected IHCs (1 to 4) and two ROIs of ISCs (III and VI) are indicated. For clarity only two IHCs are outlined by white dotted lines. **(C)** Overview of changes in Δ*F*/*F*_0_ in IHC 3 (top) and ISCs III and VI (bottom). IHC 3 showed both autonomous activity and events induced by ISC Ca^2+^ waves. The label (D) indicates an episode where IHC 3 generated bursts and ISC III and VI showed Ca^2+^ waves, which is shown at larger temporal resolution in panel (D). **(D)** Δ*F*/*F*_0_ for selected IHCs 1 to 4 and for ISC III and VI at larger time resolution. Like in control mice, IHCs of P2X2R^−/−^/P2X3R^−/−^ mice responded to ISC Ca^2+^ waves with synchronized burst-like behavior. The dotted line indicates the time point at which the image shown in (B) was taken. **(E)** IHC three generated single autonomous fCaTs (left), minibursts (≤5 fCaTs; middle) and bursts (>5 fCaTs; right). Data file: 20160824_029, sampling frequency = 32 Hz (single frame = 31.4 ms), 20×/1.0 NA objective. **(F)** Transmitted light image of the scan field in the organ of Corti of a P2X7R^−/−^ mouse aged P5 including basolateral IHC poles and adjacent ISCs at high resolution (pixel size 60 nm × 60 nm; scale bar = 10 μm). **(G)** Color-coded relative fluorescence changes Δ*F*/*F*_0_ show an exemplary Ca^2+^ wave and increased activity in most IHCs. ROIs of selected IHCs 3, 6 and 7 and one ROI of ISCs (II) are indicated. For clarity only few IHCs are outlined by white dotted lines. **(H)** Overview of changes in Δ*F*/*F*_0_ in IHC 7 (top) and ISC II (bottom). IHC 7 showed both autonomous activity and ISC Ca^2+^ wave-induced events. The label (I) indicates an episode where IHC 7 generated bursts and ISC II was part of a Ca^2+^ wave, which is shown at larger temporal resolution in panel (I). **(I)** Δ*F*/*F*_0_ for IHCs 3, 6 and 7 and for ISC II at larger time resolution. The IHCs responded to the ISC Ca^2+^ wave with synchronized burst-like behavior. The dotted line indicates the time point of the image shown in (G). **(J)** The three types of IHC Ca^2+^ signals were present in P2X7R^−/−^ mice—single autonomous fCaTs (left), minibursts (≤5 fCaTs; middle) and bursts (>5 fCaTs; right). Data file: 20170128_014, sampling frequency = 32 Hz (single frame = 31.6 ms), 20×/1.0 NA objective.

**Figure 8:**
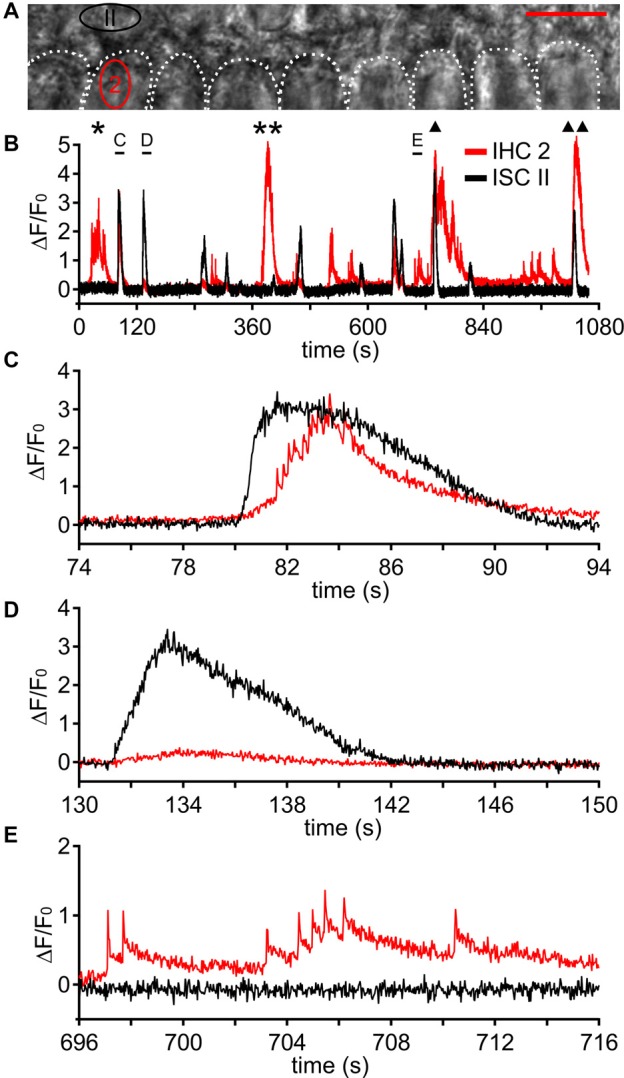
Fast Ca^2+^ transients can occur independently from ISC Ca^2+^ waves. **(A)** Transmitted light image of two rows of ISCs and the basolateral poles of nine IHCs, which are outlined by white dotted shapes; two ROIs are labeled in red (IHC 2) and black (ISC II). Scale bar = 10 μm. **(B)** Changes in [Ca^2+^]_i_ as Δ*F*/*F*_0_ in ROI “IHC 2” and in ROI “ISC II” comprising three ISCs adjacent to IHC 2 during a period of 17 min 38 s. ROI “ISC II” was invaded by several Ca^2+^ waves (black trace) whereas IHC 2 (red trace) showed bursts independent of Ca^2+^ waves from ISC II (labeled by * and **), bursts triggered by Ca^2+^ waves from ISC II (labeled by **(C)** and triangles), minibursts and single fCaTs. Three events indicated by black bars are shown at larger temporal resolution in **(C–E)**. **(C)** A Ca^2+^ wave caused a large [Ca^2+^]_i_ elevation in ISC II, which preceded the burst activity of IHC 2. **(D)** Another Ca^2+^ wave caused a [Ca^2+^]_i_ elevation in ROI “ISC II” whereas IHC 2 remained inactive. **(E)** IHC 2 showed minibursts and single fCaTs whereas the adjacent ISC II did not show any activity. Data file 20141127_002, frame rate = 34 Hz (single frame = 29.4 ms), 40×/1.0 NA objective.

**Figure 9:**
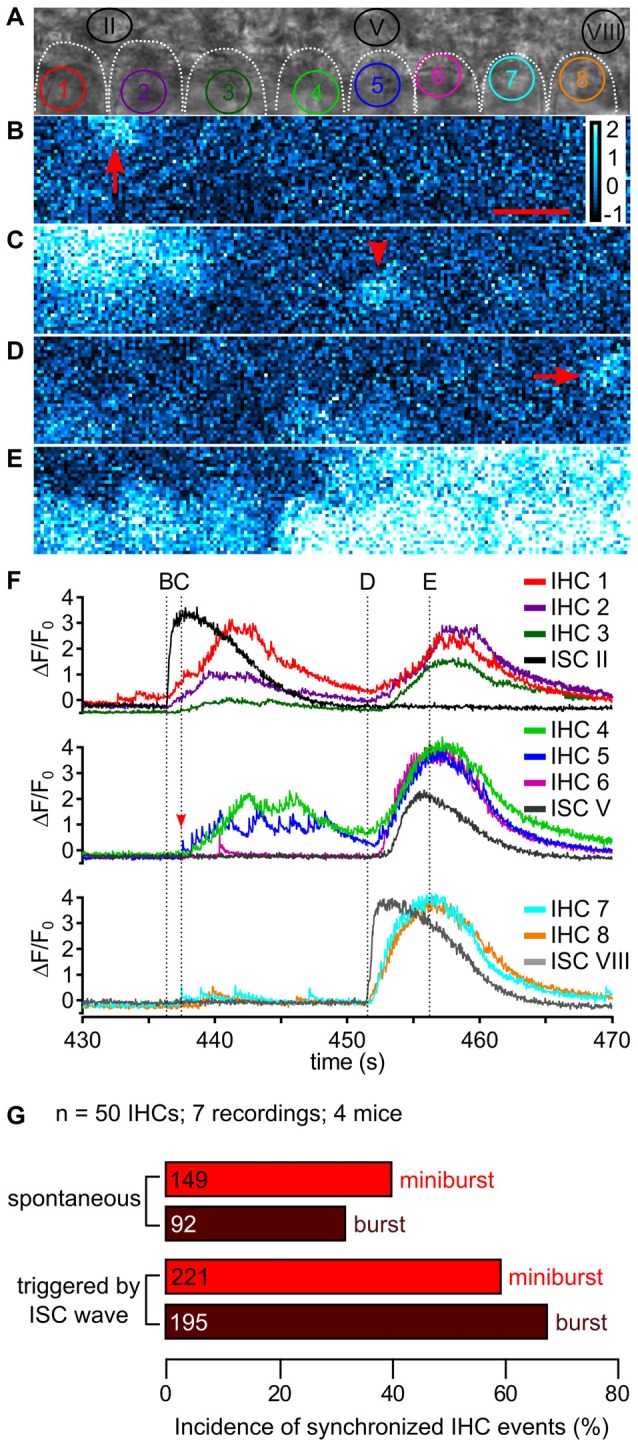
ISC activity and synchronicity of fast Ca^2+^ transients between neighboring IHCs. **(A)** Transmitted light image of the scan field in the organ of Corti of a P5 control mouse including basolateral IHC poles outlined by white dotted lines and adjacent ISCs at high resolution (pixel size: 43.2 nm × 43.2 nm). ROIs of IHCs are numbered 1 to 8; three ROIs of adjacent ISCs are indicated (II, V and VIII), with II and VIII depicting regions of wave origin, respectively. **(B–E)** Color-coded relative fluorescence changes Δ*F*/*F*_0_ at time points indicated in **(F)**. **(B)** Ca^2+^ wave one originated in ISC II (arrow). Scale bar = 10 μm. **(C)** A single isolated fCaT was generated by IHC 5 (arrowhead). **(D)** A second Ca^2+^ wave originated adjacent to IHC 8 in ISC VIII (arrow), while IHCs 4 and 5 showed elevated Ca^2+^ after burst firing. **(E)** When the ISC Ca^2+^ wave reached its maximum spread reaching out from ISC VIII to IHC 4, every IHC showed moderate (IHCs 1–3) to strong Ca^2+^ elevations (IHCs 4–8). **(F)** Δ*F*/*F*_0_ for ROIs of IHCs 1–3 (colored) and the ISC origin of wave 1 (ISC II, black, top panel); of IHCs 4–6 (colored) and ISC V adjacent to IHC 5 (dark gray, middle panel), and of IHC 7 and IHC 8 (colored) and the ISC origin of wave 2 (ISC VIII, gray, bottom). Wave 1 originating from ISC II appeared to synchronize IHCs 1–5 whereas wave 2 originating from ISC VIII synchronized all IHCs in the scan field. The Ca^2+^ increase of ISC V (middle panel, dark gray) was delayed compared with the bursts in adjacent IHCs 4–6. Vertical dotted lines and labels (B–E) in the top row refer to the time points at which images (B–E) were captured. Data file 20150114_006, frame rate = 32 Hz (single frame = 31.1 ms), 20×/1.0 NA objective. **(G)** Summary of the incidence of synchronized IHC activity occurring simultaneously in at least two adjacent IHCs as minibursts and bursts, respectively, without (top, “spontaneous”) or with an invading ISC Ca^2+^ wave as trigger (bottom). Absolute number of synchronized IHC events is given in the bars.

**Figure 10:**
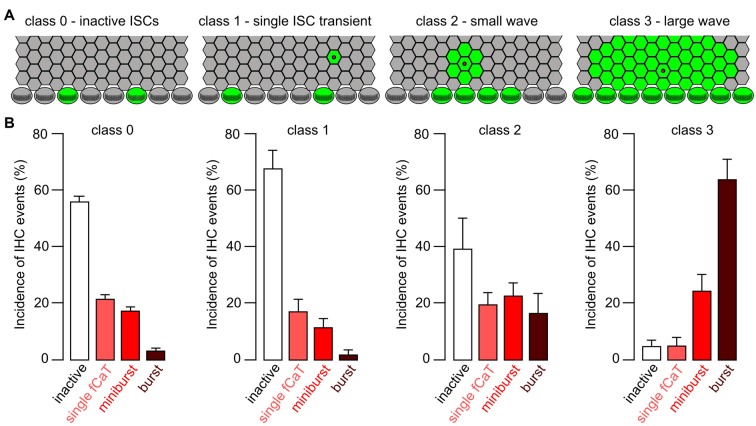
Efficacy of different classes of ISC Ca^2+^ waves in triggering fCaT activity in IHCs. **(A)** Sketch to illustrate classification of ISC activity, with a stripe of four rows of ISCs and one row of IHCs. Green color indicates elevated [Ca^2+^]_i_; wave origin is depicted by a red dot. Class 0: no ISC activity, but autonomous IHC activity; class 1: a non-propagating Ca^2+^ transient restricted to a single ISC; class 2: a small ISC Ca^2+^ wave that traveled to maximally two ISCs radially; class 3: a large ISC Ca^2+^ wave traveling >2 cells from its origin. **(B)** Average incidence ± SEM of IHC Ca^2+^ events during a defined period of time as a function of the ISC wave class. For each wave class, only the highest rank of the activity of an individual IHC was considered, in the order burst > miniburst > fCaT > inactive IHC (see “Results” section).

## Results

Using Fluo-8 AM-incubated explants of the apical organ of Corti, which were dissected with greatest care, we observed spontaneously occurring Ca^2+^ waves and corresponding waves of light refraction (crenation waves) in the region of ISCs. This is consistent with previous studies of ISCs of Kölliker’s organ in the immature organ of Corti (Tritsch et al., 2007; Anselmi et al., 2008; Dayaratne et al., 2015; Wang et al., 2015). ISCs generated diverse Ca^2+^ phenomena ranging from Ca^2+^ elevations confined to a single ISC (Ca^2+^ transients) to Ca^2+^ waves extending over various distances. Small Ca^2+^ waves propagated radially to maximum two neighboring ISC circles comprising about 200–600 μm^2^, whereas large waves covered areas from 600 μm^2^ upto >2200 μm^2^ within Kölliker’s organ involving about 75 ISCs. Sources and directions of the waves were random, indicating that the tissue had not been impaled in or near the field of view and that ATP potentially leaking out from injured or dying cells at the outermost edges of the tissue piece did not affect ISCs and IHCs in the field of view.

### Simultaneous Imaging of Ca^2+^ Waves in ISCs and Fast Ca^2+^ Transients in IHCs

Figure 2 shows a typical recording of concomitant ISC and IHC Ca^2+^ signals. Two rows of ISCs and the basolateral poles of eight IHCs are visible in the transmitted light image with the focus set at the maximum width of IHC nuclei (Figure 2A). IHCs are enwrapped by specialized ISCs, the inner phalangeal cells, which stay throughout maturity. We do not differentiate here between transient ISCs of Kölliker’s organ and inner phalangeal cells because they cannot be unambiguously discriminated in DIC images. IHCs numbered 6–8 within the Fluo-8 AM-loaded specimen showed a slightly elevated initial raw fluorescence at *t* = 0 s (green; Figure 2B). Usually care was taken to eliminate frames in which IHCs showed transient fluorescence elevations for determining the baseline (the first 10 frames) to avoid Δ*F*/*F*_0_ values <0 at later time points. Here, we show such an example on purpose to demonstrate that the calculation of relative fluorescence changes (Δ*F*/*F*_0_) eliminates different raw fluorescence values (Figures 2B,C). Figures 2C–H depict relative changes in fluorescence intensity, Δ*F*/*F*_0_ (see Eq. 1 in “Materials and Methods” section) starting at *t* = 0 s (Figure 2C) and at subsequent time points. The basolateral pole of IHC 3 showed elevated Δ*F*/*F*_0_ (Figures 2D,E, arrowheads) reflecting an autonomous single fCaT. About 1 s later, a Ca^2+^ wave originating from ISC III (Figure 2F, arrow) spread in all directions, reaching the IHCs (Figures 2G,H). fCaTs occurred in IHCs 1, 3, 4, 5, 7. Averaging Δ*F*/*F*_0_ in ROIs indicated in Figure 2A as a function of time (Figure 2I) revealed fCaTs in IHCs 3, 4 and 7, and a large and long-lasting Ca^2+^ transient in ISC III triggering the ISC Ca^2+^ wave seen at maximum spread in Figure 2H. IHC 3 was active before its adjacent ISC III generated a Ca^2+^ wave (Figure 2I, inset **i**), whereas the fCaTs in IHCs 4 and 7 started after Ca^2+^ had risen in the nearby ISC III. While IHC 7 produced 4 fCaTs shown at the onset of the recording, no elevation in Ca^2+^ could be observed in ISCs (Figure 2I, left).

### Properties and Incidence of Fast Ca^2+^ Transients in IHCs

To study the temporal properties of fCaTs in detail, IHCs were imaged at 175 Hz (scan time of a single frame: 5.73 ms), which required a smaller scan field not allowing for simultaneous imaging of ISCs. During a 573 s-long recording of eight IHCs, seven of them generated fCaTs. Single fCaTs, which were not generated within a burst of activity, had an average half-width of 32.5 ± 9.8 ms (*n* = 18) and decayed to baseline after 895.9 ± 381.2 ms (*n* = 17; Figure 3A). Time to peak, measured from 10% of the peak amplitude to the maximum fluorescence, amounted to 16.3 ± 5.5 ms (*n* = 19). Decay time defined as time from peak back to 10% above baseline was 879.7 ± 379.1 ms (*n* = 17), thus revealing a much slower decay in fluorescence compared to the rise (Figure 3A, Table 1). Besides single isolated fCaTs, IHCs generated fCaTs within events we designate as minibursts (2–5 fCaTs in close sequence, Figure 3B) and within bursts with more than five consecutive fCaTs (Figure 3C, top). The mean frequency of fCaTs was 0.32 ± 0.11 Hz, but they were not evenly distributed. Within bursts, frequencies varied from 4 Hz to 10 Hz (Figure 3C, bottom) similar to previously reported frequencies in bursts of IHC Ca^2+^ APs (Johnson et al., 2011, 2017; Sendin et al., 2014). A total of 49 bursts and 54 minibursts were generated in the seven active IHCs during 573 s recording time. Of those, 42 bursts and 18 minibursts occurred simultaneously in adjacent IHCs with at least two IHCs involved (mean number of simultaneously active IHCs: 4.62 ± 1.85). Notably, Δ*F*/*F*_0_ continuously exceeded values of 1.5 only within bursts of fCaTs (Figure 3C, top).

**Table 1:** Temporal properties of inner hair cell (IHC) fast Ca^2+^ transients (fCaTs) and average number of IHC fCaTs in minibursts and bursts.

A Properties of single fCaTs	mean ± SD	*n*
Duration (ms)	895.9 ± 381.2	17
Half width (ms)	032.5 ± 9.800	18
Time to peak (ms)	016.3 ± 5.500	19
Decay time (ms)	879.7 ± 379.1	17
**B Number of fCaTs**	**mean ± SD**	***n***
Within minibursts	02.8 ± 1.0	867
Within bursts	15.3 ± 9.5	353

***A**: Temporal properties of single fCaTs of IHCs were analyzed in recordings performed at 175 Hz scan rate. Time to peak was determined as the time between 10% and 100% of peak ΔF/F_0_. Decay time was measured as the time between 100% and 10% of the peak ΔF/F_0_ signal. **B**: Average number of IHC fCaTs in minibursts (events comprising 2–5 fCaTs) and bursts (events with ≥6 fCaTs) determined at standard scan rate (≥31 Hz)*.

To study the incidence of the three types of IHC fCaTs (single events, minibursts and bursts) perfusion was switched off in a set of experiments to exclude the possibility that the bath flow may have caused an interaction between ISCs and IHCs. Fifty IHCs from four control mice were monitored in seven recordings lasting between 544 s and 1120 s. The incidence of fCaTs was analyzed during a total observation time of 598 min (total number of IHCs multiplied by the length of respective recordings), irrespective of Ca^2+^ elevations in ISCs. IHCs generated 9668 fCaTs with 18.3% being single fCaTs, 25.2% occurring within minibursts of 2–5 fCaTs and 56.5% occurring within bursts of 6–67 fCaTs (Figure 3D). A detailed analysis showed that bursts consisted on average of 15.3 ± 9.5 fCaTs (*n* = 353) whereas minibursts contained an average number of 2.8 ± 1.0 fCaTs (*n* = 867; Table 1). During bursts of fCaTs, Δ*F*/*F*_0_ generally increased to >2 with maximal values <4 and declined slowly within 10 s. In contrast, Δ*F*/*F*_0_ reached the baseline within 1–2 s in events with ≤5 fCaTs (see Figures 3B,C). These data indicate that although the majority of fCaTs (56.5%) were embedded in a burst, a comparable number (43.5%) appeared outside of bursts as single fCaTs or as part of a miniburst (Figure 3D).

### fCaTs of IHCs Depended on External Ca^2+^ and on the Expression of Voltage-Gated Ca_v_1.3 Channels

In order to test the Ca^2+^ dependance of fCaTs, nominally Ca^2+^-free solution was applied. To achieve perfusion of the cell bodies under the reticular lamina, the application pipette here was positioned at a lateral edge of the epithelium, in contrast to the setting shown in Figure 1. When IHCs were bathed in Ca^2+^-free solution for >1 min, single fCaTs, minibursts and bursts were reversibly abolished (Figures 4A–C), indicating that influx of extracellular Ca^2+^ is required to generate fCaTs. Ca^2+^ signals of ISCs, however, did not vanish in 0 mM Ca^2+^ (Figure 4A; black trace). The decrease in the baseline of Δ*F*/*F*_0_ in both IHCs and ISCs was likely caused by a reduction in the intracellular Ca^2+^ concentration in the Ca^2+^-free solution.

Because Ca^2+^ APs of IHCs crucially depend on Ca_v_1.3 channels (Brandt et al., 2003), which account for more than 90% of voltage-gated Ca^2+^ currents in neonatal IHCs (Platzer et al., 2000), Ca^2+^ measurements were conducted in IHCs and ISCs of Ca_v_1.3^−/−^ mice in standard (1.3 mM) extracellular Ca^2+^ concentration (Figures 4D,E). In 103 IHCs from three Ca_v_1.3^−/−^ mice we did not observe a single fCaT (Figure 4E) during a total recording time of 787 min (recording length multiplied by number of IHCs). However, those IHCs contacted by an ISC Ca^2+^ wave typically showed slowly rising Ca^2+^ elevations of small amplitude (Δ*F*/*F*_0_ <1) temporally matching the Ca^2+^ signals of the ISC (Figure 4E, left). This slow IHC signal may have resulted from: (i) potential bleedthrough of the fluorescence signal of the ISCs; (ii) Ca^2+^ influx through P2X receptors; or (iii) Ca^2+^ influx through the small percentage of Ca^2+^ channels other than Ca_v_1.3 (mostly Ca_v_1.2 and some Ca_v_2.3-mediated currents; Brandt et al., 2003). We never saw this kind of small slow Ca^2+^ elevation in IHCs in the absence of an invading ISC Ca^2+^ wave. On rare occasions, IHCs of Ca_v_1.3^−/−^ mice did not respond to an invading Ca^2+^ wave with small and long-lasting Ca^2+^ elevations (Figure 4E, left). Summarizing the results from control mice in 0 mM Ca^2+^ solution and those from Ca_v_1.3^−/−^ mice in 1.3 mM extracellular Ca^2+^ we conclude that fCaTs in IHCs reflect the Ca^2+^ increases mediated by Ca_v_1.3 channels.

### Synchronized Bursts of fCaTs in Adjacent IHCs Were Triggered by ISC Ca^2+^ Waves or by Extracellular ATP

IHCs were able to generate fCaTs autonomously without being triggered by ISC Ca^2+^ waves (see Figure 3 and below). However, when an ISC Ca^2+^ wave approached the IHC region it typically evoked bursts of fCaTs in a number of adjacent IHCs (Figures 5A–C). The spontaneous Ca^2+^ wave involving ISC I (at *t* ~70 s) triggered bursts in IHCs 1 and 2, and although ISC IV was silent, IHCs 3–5 were active at the same time. The Ca^2+^ elevation of ISC I preceded that of the IHCs (Figure 5C). Analyzing 12 waves causing burst events in IHCs from a larger data set, the Ca^2+^ wave-evoked fluorescence elevation in ISCs lining the IHC row preceded the onset of fCaTs within bursts in the closest IHC by 0.52 ± 0.27 s (mean ± SD). Though less frequent, synchronized IHC bursts were also observed without any ISC activity within the recording frame as e.g., in IHCs 1, 2, 4, 5, 6, 8 at *t* ~90 s (Figure 5B and see below).

To validate previous results showing ATP-induced inward currents in neonatal IHCs (Tritsch and Bergles, 2010) in our preparation, we tested the effect of ATP on IHC fCaTs. Application of 1 μM ATP for 5 s or 10 s from the pillar side (Figure 5B) strongly increased Δ*F*/*F*_0_ in all cells of the sensory epithelium (cells of the inner and outer sulcus, interdental cells (IDCs), spiral ganglion tissue and IHCs), indicating an overall expression of purinergic receptors at the age of P4/P5. The ATP-evoked increase in Δ*F*/*F*_0_ in ISCs resembled that induced by a spontaneous Ca^2+^ wave, lasting for 10–15 s (Figure 5B; black traces). In IHCs, ATP-evoked bursts of fCaTs strongly increased Δ*F*/*F*_0_ up to 4. Repetitive ATP stimulation (five times at intervals >30 s) did not deplete the burst responses in IHCs (see Figure 5B). However, fCaTs within ATP-induced bursts were smaller in amplitude compared with fCaTs triggered by a Ca^2+^ wave and were challenging to detect (Figure 5D). Together, these experiments corroborate earlier studies showing that ATP generates Ca^2+^ waves in ISCs (Tritsch et al., 2007, 2010). To test the hypothesis if ATP can activate IHCs independently of ISC Ca^2+^ elevations, the ATP had been applied from the pillar side (Figures 5B,D,E). Such a direct stimulation of purinergic receptors on IHCs evoked bursts of fCaTs that preceded Ca^2+^ elevations in ISCs by 0.99 ± 0.34 s (*n* = 23 recordings). When taking into account that the solution flow reached the IHCs ~20 ms earlier than the ISCs, these results still indicate that ATP can elicit bursts in IHCs independent of ISC Ca^2+^ elevations.

### Block of P2 Receptors by PPADS Irreversibly Suppressed Ca^2+^ Waves in ISCs and Reversibly Reduced Fast Ca^2+^ Transients in IHCs

To further analyze the importance of purinergic signaling on fCaTs, we tested the effect of 100 μM PPADS, a broad spectrum blocker of P2Y and P2X receptors (Abbracchio et al., 2006; Coddou et al., 2011). Before PPADS application, control measurements were conducted for 180 s in order to simultaneously record Ca^2+^ waves in ISCs and single fCaTs, minibursts and bursts in IHCs (Figures 6B–D). PPADS superfusion (100 μM) completely abolished fCaTs in 33 of 44 IHCs. The remaining 11 IHCs still generated single fCaTs, but neither minibursts nor bursts. Single fCaTs, minibursts or bursts were recorded after washout in 27 IHCs, albeit with strongly reduced amplitude (Figures 6C,E). Notably, blocking P2 receptors had an even stronger effect on ISCs (Figures 6B,E): the incidence of ISC Ca^2+^ waves was reduced from 5.4 waves/100 s (35 waves in 645 s total recording time, three control mice) to 1.0 waves/100 s (five waves during 509 s in total). After removal of PPADS, the ability of ISCs to generate Ca^2+^ waves was very weak, as only three Ca^2+^ waves occurred during 1910 s washout (0.2 Ca^2+^ waves/100 s; Figures 6B–E).

### IHC fCaTs and ISC Ca^2+^ Waves Were Not Affected by the Lack of P2X2R/P2X3R or P2X7R

Because: (i) ATP strongly promoted generation of IHC bursts (Figure 5); (ii) IHCs express P2X2, P2X3 and P2X7 receptors (Brändle et al., 1999; Nikolic et al., 2003; Huang et al., 2005; Housley et al., 2006); and (iii) P2X2 and P2X3 receptors were proposed to mediate an increase in Ca^2+^ AP activity (Tritsch et al., 2007; Johnson et al., 2011), we tested the generation of fCaTs and their interplay with spontaneous ISC Ca^2+^ waves in P2X2R/P2X3R double knockout (P2X2R^−/−^/P2X3R^−/−^) and P2X7R knockout (P2X7R^−/−^) mice. Both, ISC waves and IHC fCaTs were unaffected in P2X2R^−/−^/P2X3R^−/−^ mice (Figures 7A–E). As in control mice, burst-like activity of IHCs was mostly triggered by Ca^2+^ waves and was synchronized between adjacent IHCs (Figures 7C,D). Overall, 30 IHCs from five recordings obtained from three animals, each lasting 628 s, regularly generated fCaTs. Of the 4526 fCaTs during the 314 min recording time, 665 (14.7%) were single fCaTs, 721 (15.9%) occurred in minibursts and 69.4% fCaTs were found in bursts (*n* = 3140). Examples of IHC Ca^2+^ signals from P2X2R^−/−^/P2X3R^−/−^ mice are shown in Figure 7E. Experiments in P2X7R^−/−^ mice involving 48 IHCs (five recordings, four animals) revealed similar IHCs fCaTs and ISC Ca^2+^ waves as in control mice (Figures 7F–J). Burst-like activity of neighboring IHCs in P2X7R^−/−^ mice was mostly triggered and synchronized by Ca^2+^ wave activity in close vicinity (Figures 7H,I). Of the 4648 fCaTs recorded during the total of 503 min, 1135 (24.4%) were single fCaTs, 1122 (24.1%) occurred in minibursts and 2389 (51.4%) fCaTs in bursts. Examples of IHC Ca^2+^ signals from P2X7R^−/−^ mice are shown in Figure 7J. In summary, neither P2X2, P2X3 nor P2X7 receptors were essential for the generation of IHC fCaTs nor the coupling of IHC bursts to ISC Ca^2+^ waves.

### Fast Ca^2+^ Transients Can Occur Independently of ISC Ca^2+^ Waves

It has been suggested previously that IHCs can generate Ca^2+^ APs independently of Ca^2+^ wave activity in adjacent ISCs (Johnson et al., 2011, 2017; Sendin et al., 2014). These studies are based on electrical recordings where patch pipettes could have potentially liberated ATP from injured cells at the recording site, making the interpretation of “spontaneous activity” challenging. Performing imaging only, we were able to simultaneously observe IHC fCaTs and ISC Ca^2+^ waves and their potential coupling (Figure 8). Though autonomously generated fCaTs were mostly solitary fCaTs or minibursts, spontaneous bursts were observed, too. An example is shown in Figures 8A,B where bursts of fCaTs appeared in IHC 2 without Ca^2+^ elevation in any ISC as indicated by asterisks referring to the IHC 2-trace in red. Yet, bursts with >5 fCaTs were more frequently triggered by a Ca^2+^ wave reaching the IHC region (Figure 8B, indicated by triangles, and Figure 8C) with an overall delay of 0.52 ± 0.27 s (*n* = 12 waves from four animals, see above). However, not every ISC Ca^2+^ wave elicited burst-like activity in the adjacent IHC 2 (Figure 8D). This lack of activity of IHC 2 was transient because it autonomously generated bursts (Figure 8B**) or single fCaTs and minibursts (Figure 8E) as well as ISC-evoked activity (Figure 8B, single and double triangle) thereafter. Together, our data support the hypothesis that isolated fCaTs and minibursts, but also bursts can be generated in IHCs irrespective of ISC Ca^2+^ waves.

### Simultaneous Bursts in Neighboring IHCs Were Mostly Triggered by ISC Ca^2+^ Waves

Due to their spatial spread, most Ca^2+^ waves that invaded the IHC area evoked bursts of fCaTs in more than one IHC, causing synchronous Ca^2+^ activity in two to eight IHCs. The upper number may be larger, but was limited by the size of the scan field. An example in Figure 9 shows the spatiotemporal coupling of a wave starting from ISC II, which elicited bursts in IHCs 1, 2, 3, 4 (Figures 9A–C,F). Notably, IHCs also generated simultaneous bursts without being triggered by an ISC Ca^2+^ wave. IHCs 4–8 responded with bursts to a wave starting in ISC VIII (Figure 9D), but IHCs 1–3 at 10–30 μm distance from the ISC Ca^2+^ signal also responded with bursts (Figures 9E,F) suggesting direct coupling of activity between IHCs. This means that simultaneous Ca^2+^ activity in adjacent IHCs can occur without a Ca^2+^ elevation in the ISCs contacting these IHCs or their direct IHC neighbors.

We analyzed burst activity synchronized between IHCs in periods of 15 s, the approximate duration of a large Ca^2+^ wave at a given ISC ROI, for data from four control mice (seven recordings comprising a total of 50 IHCs). The relative incidence of synchronous miniburst activity in at least two adjacent IHCs without being triggered by an ISC Ca^2+^ was 40.3% (149 cases) compared with 59.7% (221 cases) with a Ca^2+^ wave as trigger (Figure 9G). Spontaneous synchronous bursts in at least two adjacent IHCs had a relative incidence of 32.1% (92 cases), which increased to 67.9% (195 cases) when triggered by a Ca^2+^ wave (Figure 9G). A possible explanation for the coupling of Ca^2+^ activities between IHCs in the absence of Ca^2+^ waves might be an activity-driven K^+^ efflux from IHCs through their voltage-gated K^+^ channels. If an increase in the extracellular K^+^ concentration around IHCs was not effectively prevented by inner phalangeal cells/ISCs, neighboring IHCs would become depolarized, which in turn would trigger Ca^2+^ APs and hence fCaTs. A similar K^+^ accumulation-based activation of IHCs was proposed as coupling mechanism between Ca^2+^ waves and IHC activity, although the latter originates from K^+^ efflux of ISCs including inner phalangeal cells (Wang et al., 2015).

### Efficacy of ISC Ca^2+^ Waves in Triggering fCaTs in IHCs

To quantify the efficacy of a Ca^2+^ wave invading the IHC area in generating fCaTs, ISC Ca^2+^ signals were classified into four groups (Figure 10A). Wave class 0 reflects the lack of ISC activity during a 15 s period, which is the approximate duration of a large ISC Ca^2+^ wave. Class 1 defines a non-propagating Ca^2+^ transient restricted to a single ISC characterized by a mean duration of 8.5 ± 3.7 s (*n* = 12 Ca^2+^ transients). Class 2 denotes a small ISC Ca^2+^ wave that radially propagated ≤2 cells from its origin (mean duration of 10.1 ± 1.9 s, *n* = 9 waves), whereas class 3 defines a large ISC Ca^2+^ wave propagating >2 cells from its origin (mean duration of 12.5 s ± 2.0 s, *n* = 19 waves; Figure 10A). Small and large Ca^2+^ waves were only included into the analysis if they originated within the scan field. A rank order of IHC activities was defined as follows: burst > miniburst > single fCaT > inactive IHC. For a given wave class, the IHC event type with the highest rank was determined for each IHC in the scan field during the respective time window. These event numbers were added, normalized to the total number of IHCs in the scan field and averaged between recordings, yielding the relative incidence of IHC events for the respective wave class (Figure 10B).

Relating the class of ISC activity to the highest rank of the simultaneous IHC activity revealed striking differences (Figure 10B): bursts were extremely rare when ISCs were silent (4% ± 0.8%; mean ± SEM, *n* = 196 time windows without ISC activity during 15 s) or showed class 1 events (2% ± 1.5%, *n* = 13 non-propagating ISC transients). However, bursts predominated when IHCs were activated by class 3 waves. When ISCs were silent (class 0), the incidence of inactive IHCs was 57% ± 1.8% (*n* = 196) and most of the autonomous activity observed were single fCaTs (22% ± 1.4%) or minibursts (18% ± 1.3%). During a non-propagating Ca^2+^ transient of a single ISC (class 1), a similar picture emerged as with silent ISCs (Figure 10B). However, during class 2 Ca^2+^ waves (*n* = 11) the incidence of bursts increased to 17% ± 6.8% and of minibursts to 23% ± 4.5%, whereas the proportion of inactive IHCs was reduced to 40% ± 10.8%. In the presence of class 3 waves (*n* = 19), the fraction of bursts increased to 64% ± 7% and that of minibursts to 25% ± 5.7%. In contrast, the incidence of single fCaTs or inactive IHCs was reduced to 5% ± 2.8% and 5% ± 2%, respectively. These data indicate that most bursts of fCaTs reflecting burst-like action potentials of IHCs require ISC Ca^2+^ waves. On the other hand, IHCs can generate single fCaTs and minibursts without wave activity of ISCs.

## Discussion

Spontaneous activity is a hallmark of developing sensory systems including the auditory system (Blankenship and Feller, 2010; Leighton and Lohmann, 2016). Spontaneously occurring Ca^2+^ signals in IHC and their dependance on ISC Ca^2+^ waves was presently studied with Fluo-8 AM Ca^2+^ imaging on freshly dissected explants of organs of Corti. Since ATP can trigger Ca^2+^ waves in Kölliker’s organ of the immature organ of Corti (Tritsch et al., 2007; Anselmi et al., 2008; Majumder et al., 2010) and also Ca^2+^ APs in IHCs (Tritsch et al., 2007; Tritsch and Bergles, 2010; Johnson et al., 2011) it was necessary to avoid unintended release of ATP and putative ATP-dependent signaling. Great care was taken to keep the epithelium as intact as possible. Moreover, the recordings were performed from free-running tissue in the middle of the explant far from the lateral edges. By using small scan fields and a pixel size of ~0.17 μm^2^ it was possible to temporally resolve fCaTs in IHCs and classify them into single fCaTs, mini-bursts (2–5 fCaTs) and bursts of fCaTs. Our Δ*F*/*F*_0_ values of fCaTs in Fluo-8 AM-loaded IHCs varied from 0.5 up to 5 (in bursts), providing a much higher amplitude resolution than previously reported using Fluo-4 AM (Ceriani et al., 2016a).

### IHC fCaTs Are Consistent With Ca^2+^ APs

In the first postnatal week, mouse and rat IHCs generate Ca^2+^ APs that vanish shortly before the onset of hearing (Kros et al., 1998; Brandt et al., 2003, 2007; Marcotti et al., 2003b; Johnson et al., 2005, 2007, 2011, 2012, 2013; Sendin et al., 2014; Iosub et al., 2015). Ca^2+^ APs appear as single events, but more often in bursts of 10–20 single APs. They are reversibly abolished in nominally Ca^2+^-free solution (Marcotti et al., 2003b) and missing in Ca_v_1.3^−/−^ mice (Brandt et al., 2003). Similarly, the IHC fCaTs recorded in this study were reversibly suppressed in nominally Ca^2+^-free solution and absent from IHCs of Ca_v_1.3^−/−^ mice. Time-to-peak of fCaTs measured at the highest scan rate was 16 ms, which is in the range of the half-width of 15 ms for a Ca^2+^ AP at room temperature (Marcotti et al., 2003b). It is not surprising that a single fCaT lasts longer than the electrical AP because (i) Ca^2+^ influx into an IHC extends to the repolarizing phase of the AP due to the large driving force for Ca^2+^ and (ii) AP repolarization by delayed rectifier K^+^ and SK2 channels (Marcotti et al., 2003a; Mammano and Bortolozzi, 2018) is, particularly in minibursts and bursts, faster than clearance of Ca^2+^ from the cytosol by Ca^2+^ ATPases (Grati et al., 2006) or by uptake into intracellular stores and mitochondria (Kennedy, 2002), see Figure 3C. Ca^2+^-induced Ca^2+^ release from ryanodine-sensitive stores (Iosub et al., 2015) may additionally increase and prolong the Ca^2+^ signal in IHCs. Further, Fluo-8 acts as a Ca^2+^ buffer itself and could slow the Ca^2+^ decay kinetics. The dissociation time constant of the chemically related Fluo-3 is ~5 ms (Eberhard and Erne, 1989), much faster than the average decay time of fCaTs of 880 ms. Further, our fCaTs resemble the events measured with simultaneous imaging of Fluo-4FF-filled IHCs and Ca^2+^ APs recordings in current clamp (Iosub et al., 2015). Taken together, IHC fCaTs imaged under our conditions reflect the Ca^2+^ signals resulting from IHC APs.

It might be argued that due to the limited *z*-resolution of a confocal LSM compared with its resolution in *x* and *y*, we recorded signals from inner phalangeal cells rather than from IHCs, especially when IHCs were oriented steeply within the organ of Corti. However, Ca^2+^ signals of ROIs taken well inside those IHCs that had a shallow position clearly showed a different temporal pattern including fCaTs compared with adjacent ROIs likely involving inner phalangeal cells, which always lacked fCaTs (e.g., Figures 2A,I, ISC III). However, some bleed-through of fluorescence from inner phalangeal cell signals to IHCs in the *z-direction* cannot be excluded.

### ATP Independently Triggers Ca^2+^ Elevations in Both ISCs and IHCs

Applying 1 μM ATP from the pillar side towards the modiolus elicited bursts of fCaTs that were highly synchronized between neighboring IHCs and appeared before Ca^2+^ had risen in adjacent ISCs (Figure 5). This indicates that ATP can trigger IHC Ca^2+^ APs independently of ISC Ca^2+^ waves. Moreover, the large reduction of Ca^2+^ wave-independent IHC fCaTs under the broad spectrum P2R blocker PPADS (Figure 6) favors a role of P2 receptors in increasing the IHC’s susceptibility to generate Ca^2+^ APs. These results are consistent with ATP-induced inward currents through P2X receptors in IHCs (Tritsch and Bergles, 2010), which according to mRNA/protein expression data or PPADS sensitivity suggests contribution of P2X2, P2X3, P2X4, P2X7 before the onset of hearing (Nikolic et al., 2003; Huang et al., 2005; Coddou et al., 2011), yet contribution of P2Y receptors cannot be ruled out (Abbracchio et al., 2006; Huang et al., 2010). Regarding PPADS-sensitive ATP-mediated Ca^2+^ signals in ISCs, P2Y receptors such as P2Y1, P2Y2, P2Y4 and P2Y6 may be involved (Abbracchio et al., 2006; Huang et al., 2010) but P2X receptors may additionally contribute (Tritsch et al., 2007; Coddou et al., 2011).

Electrical recordings of IHC Ca^2+^ APs, which require access of the patch pipette to the body of the IHC, inevitably cause tissue damage and release of ATP, either during “cleaning” of an IHC from ISCs at one side before establishing the pipette seal, or when approaching the IHC under pressure similar as in a tissue slice. Due to local ATP release in patch-clamp experiments, the interpretation of IHC AP activity as spontaneously occurring should be taken with caution. Release of ATP also occurs when recording from ISCs (Tritsch et al., 2007; Wang et al., 2015), which makes the interpretation of the players in the interaction between Ca^2+^ waves and IHC Ca^2+^ APs and a differentiation of putative roles of ATP rather challenging. Our free-running whole-mount preparation has the advantage that the epithelium was left intact within a region much larger than the field of view, which allowed imaging the interaction of ISC Ca^2+^ waves and IHC fCaTs without local tissue damage and contamination with ATP. The fact that ISC Ca^2+^ waves did not repeatedly originate from the same foci suggests that the tissue in the field of view was not injured.

### Electrical Properties of IHCs Sustain Autonomous Ca^2+^ AP Activity

Frequently, single fCaTs and minibursts but sometimes also bursts were observed in IHCs in the absence of Ca^2+^ waves in adjacent ISCs (Figures 5, 8, 9; summarized in Figure 10), which were termed here spontaneous or autonomous IHC events. These events reflecting Ca^2+^ APs were therefore neither the consequence of extracellular K^+^ accumulation through K^+^ efflux nor of ATP release from the ISCs (see below). It is rather likely that sizes and properties of IHC currents, such as Ca_v_1.3 currents, various K^+^ currents including SK2, transducer currents at rest, purinergic currents, cholinergic currents (Brandt et al., 2003; Marcotti et al., 2004; Housley et al., 2006; Johnson et al., 2007, 2012) favor to the ability of the IHC to produce regenerative Ca^2+^ APs.

### Coupling of ISC Ca^2+^ Waves to IHC Ca^2+^ Signals

When an ISC Ca^2+^ wave invaded the IHC region it typically elicited bursts of fCaTs in IHCs. The sizes of ISC Ca^2+^ waves correlated with the number of activated IHCs; large waves activated more IHCs that responded by synchronously generating bursts with an average delay of 0.52 s (Figure 10). The mechanism of coupling between ISCs and IHC APs is not entirely clear. One proposed mechanism is the regenerative efflux of ATP through connexin hemichannels into the endolymph and activation of P2X2 and P2X3 receptors (perhaps also P2X7) at the IHC (Anselmi et al., 2008; Majumder et al., 2010; Johnson et al., 2011; Mammano and Bortolozzi, 2018). As the ATP superfused from the pillar side evoked bursts in IHCs *before* eliciting Ca^2+^ transients in the ISCs (Figures 5D,E), a direct ATP activation of IHCs could possibly occur *in vivo*. However, the fact that IHC fCaT activity was unaffected in P2X2R^−/−^/P2X3R^−/−^ and in P2X7^−/−^ mice indicates that neither of the receptors was essential for activating IHCs by incoming Ca^2+^ waves. Because IHCs from P2X4R^−/−^ mice produced normal Ca^2+^ APs (Sendin et al., 2014), an essential role of P2X4 receptors for IHC Ca^2+^ AP activity can be excluded, too. Recently, an alternative, ATP-independent mechanism of IHC activation has been proposed, in which the Ca^2+^ elevation in ISCs opens Ca^2+^-dependent Cl^−^ channels resulting in a coupled efflux of Cl^−^, K^+^ and water (Wang et al., 2015). The accumulation of K^+^ ions around the IHCs in turn depolarizes the IHC over the threshold of AP generation (Wang et al., 2015).

### Does AP-Induced Extracellular K^+^ Accumulation Synchronize Neighboring IHCs?

We repeatedly observed that neighboring IHCs synchronously generated Ca^2+^ signals (from single fCaTs to bursts) in the absence of ISC Ca^2+^ waves (Figure 5A,B, 9E). Because AP activity of an IHC itself causes efflux of K^+^ ions (Marcotti et al., 2003a, 2004), a sizeable amount of K^+^ ions may have been able to depolarize neighboring IHCs over the threshold for generating Ca^2+^ APs. The basolateral part of an IHC is surrounded by specialized ISCs, the inner phalangeal cells, leaving a small extracellular volume in which K^+^ efflux from the IHC may lead to a substantial increase in the extracellular K^+^ concentration. Inner phalangeal cells around the IHCs normally remove K^+^ ions by means of Kir4.1 channels (Eckhard et al., 2012), but their uptake rate might be insufficient at times, leading to a series of fCaTs in an IHC and its neighbors.

### Spontaneous Activity in the Immature Cochlea

The limited number of fCaTs in a burst (mean: 15.3 ± 9.5) and its limited duration suggest that a rising Ca^2+^ level probably activated SK2 channels, which in turn hyperpolarized the membrane potential of the IHCs and prevented further Ca^2+^ APs (Johnson et al., 2007), shaping a temporally structured pattern of Ca^2+^ APs (Johnson et al., 2011; Sendin et al., 2014). *In vivo*, AP patterning in the central auditory system is additionally shaped by inhibition of IHC Ca^2+^ AP activity through efferent cholinergic fibers acting via α9/α10 acetylcholine receptors coupling to SK2 channels (Glowatzki and Fuchs, 2000; Clause et al., 2014). Our data support the existence of two independent types of spontaneous activity in the developing auditory system, autonomous generation of Ca^2+^ APs in IHCs independent of ISC Ca^2+^ waves and the stochastic generation of Ca^2+^ transients in ISCs that elicit Ca^2+^ waves inducing synchronized bursts of IHC Ca^2+^ APs. The role of these different types of spontaneous activity for the firing patterns of spiral ganglion neurons with a functioning inhibitory efferent system *in vivo* needs to be established.

## Author Contributions

JE and TE designed the study. TE and KB acquired Ca^2+^ imaging data. TE, KB and IM analyzed the data. TE, KB, IM and JE prepared the manuscript.

## Conflict of Interest Statement

The authors declare that the research was conducted in the absence of any commercial or financial relationships that could be construed as a potential conflict of interest. The reviewer RL and handling Editor declared their shared affiliation at the time of review.
